# Understanding the Molecular Genetics of Basal Cell Carcinoma

**DOI:** 10.3390/ijms18112485

**Published:** 2017-11-22

**Authors:** Cristina Pellegrini, Maria Giovanna Maturo, Lucia Di Nardo, Valeria Ciciarelli, Carlota Gutiérrez García-Rodrigo, Maria Concetta Fargnoli

**Affiliations:** Department of Dermatology, Department of Biotechnological and Applied Clinical Sciences, University of L’Aquila, 67100 L’Aquila, Italy; cristina.pellegrini@cc.univaq.it (C.P.); mariagiovanna.maturo@graduate.univaq.it (M.G.M.); luciadinardo@hotmail.it (L.D.N.); valeria.ciciarelli@hotmail.it (V.C.); carlota.gutierrez.gr@gmail.com (C.G.G.-R.)

**Keywords:** basal cell carcinoma, molecular genetics, *PTCH*1, *TP*53, *MYCN*, *PTPN*14, *LATS*1, *TERT* promoter, *DPH*3 promoter

## Abstract

Basal cell carcinoma (BCC) is the most common human cancer and represents a growing public health care problem. Several tumor suppressor genes and proto-oncogenes have been implicated in BCC pathogenesis, including the key components of the Hedgehog pathway, *PTCH*1 and *SMO*, the *TP*53 tumor suppressor, and members of the *RAS* proto-oncogene family. Aberrant activation of the Hedgehog pathway represents the molecular driver in basal cell carcinoma pathogenesis, with the majority of BCCs carrying somatic point mutations, mainly ultraviolet (UV)-induced, and/or copy-loss of heterozygosis in the *PTCH*1 gene. Recent advances in sequencing technology allowed genome-scale approaches to mutation discovery, identifying new genes and pathways potentially involved in BCC carcinogenesis. Mutational and functional analysis suggested *PTPN*14 and *LATS*1, both effectors of the Hippo–YAP pathway, and *MYCN* as new BCC-associated genes. In addition, emerging reports identified frequent non-coding mutations within the regulatory promoter sequences of the *TERT* and *DPH*3*-OXNAD*1 genes. Thus, it is clear that a more complex genetic network of cancer-associated genes than previously hypothesized is involved in BCC carcinogenesis, with a potential impact on the development of new molecular targeted therapies. This article reviews established knowledge and new hypotheses regarding the molecular genetics of BCC pathogenesis.

## 1. Introduction

Basal cell carcinoma (BCC) is the most common malignant neoplasm in fair-skinned individuals, and accounts for about two-thirds of all skin cancers in Caucasians [1]. The incidence reached 2.75 million cases worldwide [2], representing a growing public health care problem. BCC rarely metastasizes or causes death, but it can result in extensive morbidity through local invasion and tissue destruction [1]. The appearance of BCC is strongly associated with exposure to ultraviolet (UV) radiations: tumors develop primarily on the sun-exposed skin of elderly individuals with fair skin type; they are rarely found on palmo-plantar surfaces or in children, and never appear on the mucosa. Men are more frequently affected than women, usually after the age of 50. However, a remarkable increase in the incidence of BCC has been recently observed in young women, probably due to a higher use of tanning beds for cosmetic purposes and to a closer attention to their appearance and the health of their skin [3]. Younger individuals develop BCC predominantly located on the trunk and limbs, caused by increasing affluence and consequent leisure-related, episodic sun exposure [4]. Additionally, established risk factors of BCC include ionizing radiations, arsenic ingestion, and immune suppression [5].

Evidences of genetic alterations in BCC pathogenesis derive from studies of patients with a hereditary predisposition syndrome, known as basal cell nevus syndrome (BCNS) or Gorlin syndrome. As early as 1894, Jarisch and White first described patients with clinical features of the autosomally inherited BCNS syndrome [6,7] which was later characterized in detail by Gorlin and Goltz [8]. BCNS patients typically develop multiple BCCs starting at a young age and are prone to develop other tumors including medulloblastomas. Using family-based linkage studies of kindreds with BCNS, the locus carrying the causative mutant gene was mapped to human chromosome 9q22 and then to the Patched 1 (*PTCH*1) gene [9]. PTCH1 is a transmembrane receptor that acts negatively in the hedgehog (HH) signaling pathway. After the identification of *PTCH*1 as the BCNS disease gene [10,11], *PTCH*1 and other components of the HH pathway, as smoothened (*SMO*) and glioma-associated oncogene (*GLI*), were investigated in sporadic BCC [12,13,14]. These pioneering studies demonstrated that loss-of-function mutations of *PTCH*1 and/or gain-of-function mutations of *SMO* were implicated in the pathogenesis of this disease. In subsequent reports, including a high number of sporadic BCCs, molecular alterations of the HH pathway components were identified in about 90% of the cases [12,14,15,16,17,18,19]. Therefore, the aberrant HH signaling activation was defined as a prerequisite for the development of BCC both for the inherited (Gorlin’s syndrome) and for the sporadic forms of the disease.

Beyond HH signaling, other tumor suppressor genes and proto-oncogenes have been implicated in the pathogenesis of BCC, including the *TP*53 tumor suppressor gene and members of the *RAS* proto-oncogene family [17,18,19,20,21]. In a recent study including 293 BCC tumors, the driver pivotal role of *PTCH*1, *TP*53, and *SMO* has been confirmed; however, 85% of BCC also harbored additional driver mutations in other cancer-related genes, such as *MYCN*, *PPP*6*C*, *PTPN*14, *STK*19, and *LATS*1 [22]. Finally, emerging reports have identified somatic mutations within regulatory sequences as the promoters of the telomerase reverse transcriptase (*TERT*) gene and of the diphthamide biosynthesis 3 (*DPH*3) gene [23,24,25,26].

Of note, the mutational pattern of genes involved in BCC tumorigenesis is consistent with UV-induced DNA damage, since genes harbor “UV signature” mutations [27]. Solar radiation (UVB and UVA) can mutagenize DNA, producing UV landmark C to T or CC to TT transversions via cyclobutane dimers and pyrimidine(6–4)pyrimidine photoproducts [28]. The transformation of the keratinocytes occurs when these mutations affect the function of multiple oncogenes, tumor-suppressor genes and important housekeeping genes, leading to an unregulated cell cycle [29].

This review aims to provide an overview of the molecular genetics of BCC pathogenesis, describing the mutational events occurring both in established and recently discovered BCC driver genes.

## 2. Established BCC-Associated Genes

### 2.1. HH Pathway Genes

HH is a highly-conserved development pathway involved in organogenesis, stem cell maintenance, tissue repair, and regeneration. In the skin, the HH pathway is responsible for maintaining the stem cell population and for controlling development of hair follicles and sebaceous glands [30]. Aberrant HH pathway activation controls multiple aspects of tumorigenesis including initiation, progression, and relapse, at least in part, by driving a cancer stem cell phenotype [31].

**Figure 1:**
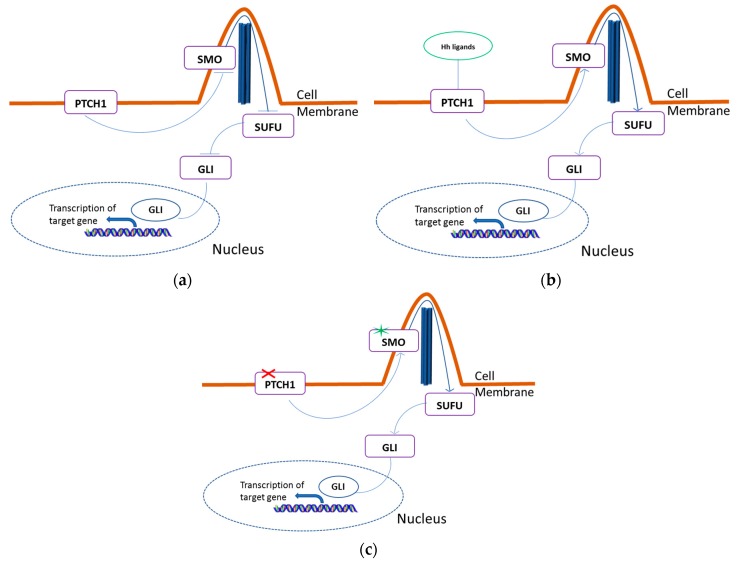
Physiologic and oncogenic Hedgehog signaling. (**a**) In the absence of HH ligands, *PTCH*1 constitutively represses *SMO*, blocking the HH signal transduction; (**b**) The family of extracellular HH ligands binds to *PTCH*1, de-represses *SMO* thereby allowing its translocation on the tip of the primary cilium. *SMO* sends signals through a series of interacting proteins, including *SUFU*, resulting in activation of the downstream *GLI* family of transcription factors; (**c**) Loss of function of *PTCH*1 (red cross) or activating mutations of *SMO* (blue asterisk) induces HH pathway in the absence of HH ligands. HH; Hedgehog; *PTCH*1; Patched Homolog 1; *SMO*; *SMO*othened; *SUFU*; suppressor of fused; *GLI*; glioma-associated oncogene.

The canonical HH pathway contains several key components, including the HH ligands as sonic HH, Indian HH, and Desert HH, the transmembrane receptor proteins *PTCH*1 and *PTCH*2, the G protein coupled receptor-like protein *SMO,* and the *GLI* transcription factors 1, 2, and 3 (*GLI*1, *GLI*2, and *GLI*3) [32] (Figure 1). HH signaling transduction depends on the primary cilium structure, a highly specialized microtubule-based organelle that protrudes from the plasma membrane in almost all cell types and acts as a sensor for extracellular signals [33,34]. The pathway is activated when HH ligands bind *PTCH*1 to relieve *PTCH*-mediated *SMO* inhibition at the base of primary cilium [35]. *SMO* then translocates to the cilium, driving a signaling cascade that leads to release of the *GLI* proteins, sequestered in the cytoplasm by several proteins, including the suppressor of fused (*SUFU*). Then, *GLI* transcription factors translocate into the nucleus and activate transcription of context-specific genes regulating self-renewal, cell fate, survival, and angiogenesis. In addition, *GLI*1 establishes a feedback loop that auto-regulates HH signaling through *PTCH*1 modulation [36]. Mutations at any level of the HH signaling pathway (e.g., *PTCH*1, *SMO*, and *SUFU*) will result in an increased expression of *GLI*1.

In addition to the abovementioned signaling pathway, there is a non-canonical HH cascade resulting in the activation of *GLI* transcription factors independent of HH ligands or *PTCH*1/*SMO*. *GLI* activity has been shown to be regulated positively by KRAS, TGF-β, PI3K-AKT, and PKC-α [37,38,39,40,41,42,43], and negatively by p53, PKA, and PKC-δ [43,44,45,46].

Upregulation of HH signaling represents the most significant pathogenic event in BCC. More than 90% of BCCs show a loss of *PTCH*1 function by inactivating *PTCH*1 mutations, as well as by aberrant activation of *SMO* through activating *SMO* mutations [12,14,15,16,17,18,19,47,48,49,50].

The *PTCH1* gene has been mapped to 9q22.3 and consists of 23 exons spanning approximately 74 kb encoding a 1447 transmembrane glycoprotein. The sequence suggests that *PTCH*1 is a transmembrane protein with 12-membrane spanning domains and 2 large extracellular loops. The inactivation of *PTCH*1 in BCCs may be a necessary, if not sufficient, event for carcinogenesis. Sporadic BCCs have been reported to carry inactivating point mutations, copy loss of heterozygosity (LOH), and copy-neutral LOH (due to uniparental disomy) in *PTCH*1 [19,49,50]. *PTCH1* somatic mutations range between 11% and 75% (Table 1) [11,12,15,18,19,22,48,49,50,51,52] and are represented by non-synonymous mutations with a predominance of nonsense and splice site mutations throughout the entire length of the *PTCH*1 gene, without evidence for a hot-spot region. About half of these mutations contains the “UV-signature” C-T and tandem CC-TT transitions [17,18,19,22,48,51,53]; however, the UV radiation origin of *PTCH*1 mutations is controversial, since other factors, such as oxidative stress, have been implicated in the mutagenesis of this gene [12,18,22,54]. Besides point mutations, somatic copy number aberrations (SCNAs) of *PTCH*1 have been frequently reported in BCC [12,15,18,19,22,48,49,50,51,52]. LOH of the *PTCH*1 allele is the most frequently identified SCNA in BCC and occurs in about 42–70% of the tumors due to loss of part of or the whole of 9q chromosome arm [19,55,56] (Table 1).

Activating *SMO* mutations were found in 10–20% of sporadic BCCs and are mainly represented by missense changes affecting codon 535 [14,19,22,62]. Functional studies on the W535L variant demonstrated that it is a constitutively active mutant and significantly increases basal HH activity in the absence of HH ligand [62]. Recent studies revealed that up to 8% of BCCs carry loss of function *SUFU* variants, both missense and nonsense mutations, which are able to disrupt its binding to *GLI,* thus leading to constitutive pathway activation [19,22,48]. A higher frequency of *SUFU* mutations has been reported by Urman et al. [63], although it is considered as a likely passenger mutation. Finally, the homologue *PTCH2* gene, which shows a 57% of similarity with *PTCH1* and also serves as a receptor, has been found to carry mutations in a small number of sporadic BCC [64,65].

### 2.2. TP53 Gene

The second most frequent event associated with BCC pathogenesis is the inactivation of the *TP*53 gene. The *TP*53 tumor suppressor gene is involved in cell cycle arrest and activation of programmed cell death [66,67]. As a guardian of the genome, *TP*53 is stabilized upon stress by phosphorylation and alters the expression of different sets of downstream target genes including those that cause cell cycle arrest [66,67,68]. In a mouse model investigating BCC pathogenesis, loss of *TP*53 has been shown to upregulate the activity of the HH pathway by increasing *SMO* expression and rendering the mouse interfollicular keratinocytes receptive for X-ray induced BCCs [69].

Inactivating *TP*53 genetic alterations have been detected in 50% of human cancers, including all skin carcinomas, which are believed to be a very early if not initial event in carcinogenesis [70,71]. In skin cancers, the majority of *TP*53 missense substitutions are located in the central DNA-binding core domain (codons 102–292) and include codons 177, 196, 245, 248, 278, and 282, producing a full-length protein with altered function [57,58,59,60,61,71]. Regarding BCC, non-synonymous mutations in the *TP53* gene have been reported in about half of sporadic cases whereas LOH has been described with a much lower frequency in BCC as compared to other tumors as colon, lung, and bladder cancers [57,58,59,60,61] (Table 1). Hot spots occurring specifically in BCC have been found at codons 177, 196, and 245 [58,71]. Codon 177 seems to be specific for BCC since it is not frequently mutated in other malignancies. Little is known about this codon but it is interesting to note that it includes a sequence slowly repaired after UV-irradiation [72]. Both codons 196 and 245 have been found to be mutated in breast and colon cancers. Codon 245 seems to play a major role in carcinogenesis being implicated in several tumor types, such as lung, head and neck, ovary, stomach, and esophagus malignancies [58,71].

The majority of *TP*53 mutations in BCC are C to T transitions, with a high frequency of CC to TT double base changes, clearly indicative of UV-induced changes [17,57,58,72]. A lower level of *TP*53 mutations were indeed identified in BCCs from sunscreen users compared to that of non-sunscreen users [59].

## 3. Novel BCC-Associated Genes

Recent improvements in technologies for genomic analysis have led to the identification of new driver genes for BCC, featuring a more complex genetic network of cancer-associated genes than previously hypothesized. It is noteworthy that there are discrepancies regarding the list of driver genes identified across the different studies, probably reflecting the clinico-pathological heterogeneity of analyzed BCCs, e.g., BCCs with a low risk or a high risk of recurrence, tumors from Gorlin syndrome patients, or BCCs naïve or resistant to target therapy [19,22,26,48].

### 3.1. Hippo-YAP Signaling Genes

**Figure 2:**
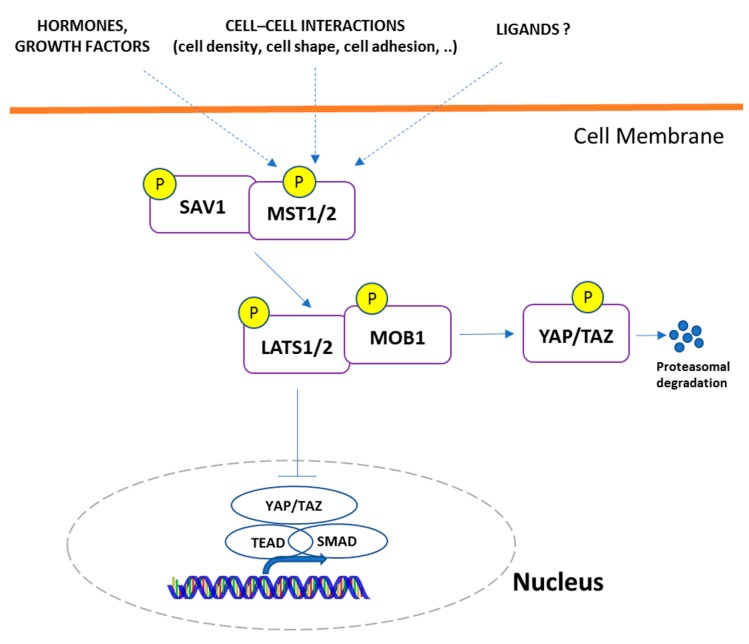
Hippo–YAP pathway. When the Hippo pathway is activated, MST1/2 kinases and SAV1 form a complex to phosphorylate and activate LATS1/2 and MOB1. Activated LATS1/2 phosphorylates YAP/TAZ, that is sequestered in the cytoplasm or degraded. Dephosphorylation of YAP/TAZ allows its traslocation into the nucleus and the interaction with TEAD1-4 to induce the expression of genes promoting tumor progression. MST, mammalian Ste2-like kinases Hpo orthologs; SAV, Protein Salvador Homolog 1; LATS, Large Tumor Suppressor Kinase Wts orthologs; MOB1, Mob-as-tumor-suppressor homologs; YAP, Yes Associated Protein Yki ortholog; TEAD, transcriptional enhancer associate domain; TAZ, transcriptional co-activator with PDZ-binding motif; SMAD, Mothers against decapentaplegic homolog.

The Hippo pathway is crucial in organ size control, and its deregulation contributes to tumorigenesis (Figure 2). This pathway was initially investigated in *Drosophila*, in which mosaic mutations of Hippo-related genes resulted in tissue overgrowth [73,74]. The Hippo axis includes a series of kinases that, through a cascade of phosphorylation events, inactivate the transcriptional co-activator Yes-associated protein (YAP), thus regulating cell proliferation and apoptosis [75]. The YAP1 protein is the major downstream effector of the Hippo pathway [74] and the kinases MST1/2 and LATS1/2 represent the core component of the mammalian Hippo signaling. MST1/2 phosphorylate and activate LATS1/2 kinases, preventing the translocation of YAP1 and its family member TAZ (trascriptional co-activator with PDZ-binding motif) into the nucleus [75,76]. YAP1 transactivation may be inhibited by the non-receptor tyrosine phosphatase 14 (PTPN14) that promotes its nucleus-to-cytoplasm translocation through LATS1 activation [77,78].

Many components of the Hippo–YAP cascade have been found to be deregulated in human cancers [75,79,80], and, more recently, also in BCC. Genetic studies in mouse models showed that this pathway acts in balancing cutaneous growth and differentiation [81] and that elevated nuclear YAP1 levels lead to massive expansion of proliferative basal epidermal cells [80].

Aberrant LATS1 and PTPN14 function may represent an independent mechanism for activation of Hippo–YAP pathway. *LATS*1 gene has been investigated in only one study reporting inactivating mutations in 47 of 293 (16%) BCCs, with 24% of the mutations being truncating, consistent with the tumor suppressor role of *LATS*1 [22]. The most frequent mutation was the R995C, occurring in the core of the kinase domain. Interestingly, in a previous case report, a Japanese patient with Gorlin syndrome was shown to carry a biallelic inactivation of *LATS*1 in an infiltrative BCC [82]. In addition to *LATS*1 mutations, missense mutations in the *LATS*2 gene, a paralog of *LATS*1, were reported in 12% of BCCs and in the *PTPN*14 gene in 23% of BCCs with 61% truncating changes [22]. In line with the identification of *LATS*1 and *PTPN*14 mutations, RNA sequencing studies showed that the Hippo–YAP pathway is significantly upregulated in BCC [22].

### 3.2. MYCN/FBXW7 Signaling

MYCN is a member of the MYC family of transcriptional activators and a potential downstream effector of the HH pathway [83]. Changes in the levels of MYC family transcription factors profoundly influence cell growth, proliferation, differentiation, and apoptosis [84]. *MYCN* missense mutations have been identified in 30% of BCCs [22], with most of the mutations mapping in the region encoding the MYC box 1 domain, which is involved in the interaction with FBXW7 tumor suppressor [22]. FBXW7 is a component of the SCFFbw7 ubiquitin ligase that promotes proteasome-dependent MYC degradation through the ubiquitin pathway [84]. Functional studies demonstrated that four of the most frequent N-MYC substitutions found in BCC, T58A, P59L, P60L, and P63L impaired the binding with the FBXW7, resulting in excessive amounts of the N-MYC protein [22]. Aberrant copy-gain rarely occurs in BCC, while gene amplification is the main mechanism of pathogenic up-regulation of MYCN in medulloblastoma and neuroblastoma [22,83,85]. Interestingly, deleterious mutations and LOH events in the *FBXW*7 gene occur in 5% and 8% of BCCs samples, respectively, suggesting a selective pressure for enhanced N-MYC stability in BCC [22].

### 3.3. TERT-Promoter

*TERT* gene encodes the catalytic reverse transcriptase subunit of telomerase that maintains telomere length. Increased telomerase activity is perceived to be one of the hallmarks of human cancers, and the transcriptional regulation of the *TERT* gene is the major cause of its cancer-specific activation [86].

**Figure 3:**
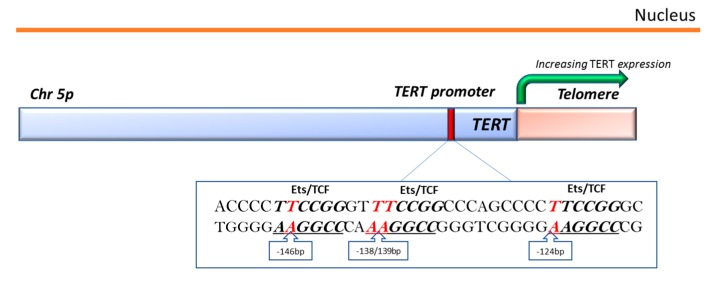
Schematic representation of *TERT* promoter structure. *TERT* promoter region (red bar) and *TERT* gene (pink bar). Core consensus sequence with Ets/TCF regulatory elements of *TERT* promoter and location of BCC-specific mutations (red) are reported in the box. The Ets/TCF binding motifs for Ets/TCF transcription factors created by mutations are underlined. *TERT*, telomerase reverse transcriptase; Ets/TCF; Ternary complex factor (TCF) subgroup of the Ets E26 transformation-specific transcription factor.

The *TERT* gene is located on chromosome 5p15.33 and the promoter region of this gene is considered the most important regulatory element for telomerase expression (Figure 3). The core promoter region consists of 260 base pairs with several binding sites for transcription-factor that regulate gene transcription [87]. *TERT* promoter mutations have been detected at a high frequency in many different cancers as melanoma, non-melanoma skin cancers, bladder cancer, and glioma. They have been related to increased TERT expression through de novo creation of binding sites for Ets/TCF transcription factors, higher telomere length, and with markers of poor outcome [86,88]. The high recurrence, specificity, and gain of function of non-coding promoter *TERT* mutations support that they are driver rather than passenger events in cancer development [89].

The discovery paper reported a disease-segregating causal germline mutation in a large melanoma family at −57 bp position from the ATG start site and recurrent somatic mutations at −124 and −146 bp positions in tumors from unrelated patients [90] (Figure 3). A few studies have recently investigated *TERT* promoter in BCC tumors, identifying a high prevalence of mutations. Most of *TERT* promoter mutations exhibit an UV-signature with C>T or CC>TT changes, again supporting an etiologic role for UV exposure [23,24,25].

### 3.4. DPH3-OXNAD1 Bidirectional Promoter

Similar to *TERT*, recurrent mutations in noncoding positions close to the transcription start site have been reported in the bidirectional promoter of both *DPH3* and oxidoreductase NAD-binding domain containing 1 (*OXNAD*1) genes. DPH3 is required for the synthesis of diphthamide, a modified histidine residue in eukaryotic translation elongation factor 2 that helps in the maintenance of translation fidelity. DPH3 silencing impairs in vivo metastasis in mouse melanoma cells, and its family member DPH1, which is also required for diphthamide synthesis, has been attributed a tumor-suppressor role [91].

Mutations in the bidirectional promoter region of *DPH3-OXNAD*1 (called *DPH*3 promoter) were first described in melanoma in two whole genome screenings for mutations in the regulatory regions of the genome [92,93]. The reported frequency of *DPH*3 mutations in melanoma was 16% (6/38) and 29.4% [92,93]. Typical UV signature mutations of the *DPH*3 promoter were recently shown to be common in BCC (42%) and squamous cell carcinoma (39%) [26]. Mutations occurred at sites adjacent and within a binding motif for the Ets/TCF transcription factor, at −8 and −9 bp from *DPH*3 transcription start site. Reporter assays carried out in one melanoma cell line for *DPH*3 and *OXNAD*1 orientations showed statistically significant, increased promoter activity due to −8/−9 CC > TT tandem mutations, although no effect was observed on *DPH*3 and *OXNAD*1 transcription in tumors [26].

### 3.5. Other Potential BCC-Associated Genes

A high frequency of mutations significantly associated with BCC tumorigenesis was observed in two cancer-related genes, *PPP*6*C* and *STK*19, in a recent study, including a large cohort of tumors [22]. *PPP*6*C* regulates cell cycle progression in human cells through control of cyclin D1 and inactivation of RB1 and participates to LATS1 activation [94,95]. Mutations were detected in 15% of BCC, with the most frequent being the R264C substitution, shown to impair the phosphatase activity of the encoded protein [96]. The *STK*19 gene encodes a kinase with an unknown function probably involved in transcriptional regulation and was found to be mutated in 10% of BCC tumors across the entire length of the gene. The most recurrent change was the D89N substitution, a mutation previously described in 5% of melanomas [97].

Other candidate cancer genes were found to be frequently mutated in BCC (Table 1), including *ARID*1*A*, *CASP*8, *CSMD*1, *GRIN*2*A*, *KRAS*, *NOTCH*1, *NOTCH*2, *NRAS*, *PIK*3*CA*, *PREX*2, and *RAC*1, although they did not show a statistically significant association with BCC [19,22]. Among them, two independent exome-sequencing studies [19,22] reported a high frequency of mutations in *NOTCH1* (29% and 50%, respectively) and *NOTCH2* (26% and 67%, respectively) genes, mostly relating to loss of function, thus suggesting their tumor-suppressor role in BCC. Missense and truncating mutations in both genes have been detected across the entire length of the genes, without any evidence of hot-spot changes.

## 4. Molecular Therapy

The identification of tumor-specific genetic alterations is currently one of the most active areas of cancer research and is having a major impact on the development of novel therapies targeting known deregulated signaling pathways.

Aberrant activation of the HH pathway is the key molecular alteration in BCC growth and progression; thus, recent research has focused on developing therapeutic strategies targeting this signaling cascade. Two HH pathway inhibitors, vi*SMO*degib and sonidegib, both targeting the *SMO* protein, are currently FDA- and EMA-approved for treatment of advanced BCC. Other *SMO* inhibitors, as LY3039478 (Taledigib), IPI-926 (saridegib), and PF-04449913 (glasdegib), are currently being investigated in clinical trials in advanced solid tumors, including BCC [98,99,100]. The majority of BCC patients treated with the *SMO* inhibitors experience clinical benefits [101,102,103], although acquired resistance has been reported [104,105,106]. Resistance occurs predominantly through de novo mutations of the drug target *SMO* and, to a lesser extent, through concurrent copy number changes in *SUFU* and *GLI*2 [107] or upregulation of synergistic signals such as PI3K pathway [108]. For patients with acquired resistance to *SMO* inhibitors, possible solutions may include pharmacological targeting of effectors downstream of *SMO* or combination strategies with other molecular targeted therapies (e.g., PI3K, EGFR inhibitors) [106].

Small molecules targeting the *GLI* transcription factors, defined as *GLI* antagonists, are an emerging cancer therapy currently under investigation in preclinical studies and might be effective in tumors with canonical and non-canonical activation of HH pathway [106]. Verteporfin, a second-generation photosensitizer approved by the FDA for the treatment of age-related macular degeneration, has been shown to inhibit the proliferation of hepatocellular carcinoma and retinoblastoma cells by inhibiting the YAP pathway [109,110]. Bakshi et al. [111] are currently investigating the effects of verteprofin administered as monotherapy and in combination with *SMO* inhibitors in BCC tumor regression.

Monoclonal antibodies that block immune checkpoint proteins, as anti- programmed cell death-1 (PD-1) and PD-Ligand-1 (PD-L1), thus enhancing the anti-tumor immune response, have demonstrated remarkable efficacy against multiple cancers, including melanoma [111]. Several immune-related markers have been implicated in BCC pathogenesis [112,113,114,115,116,117], suggesting that immunotherapy may be a valuable therapeutic option for this tumor. Recently published case reports provide evidence for the efficacy of anti-PD-1 therapy in patients with advanced BCC, either as initial treatment or after acquired resistance to HH pathway inhibition [118,119,120]. In addition, a phase I clinical trial is currently investigating if pembrolizumab (anti-PD1) can be used with or without vismodegib to treat metastatic or unresectable BCCs (NCT02690948) [111].

## 5. Conclusions

Aberrant activation of the HH pathway is the hallmark of BCC carcinogenesis, with the majority of BCCs harboring *PTCH*1 and, less frequently, *SMO* mutations. Recent genomic studies discovered additional signaling pathways associated with the development of BCCs. The identification of inactivating mutations in two key components of the Hippo–YAP pathway, *LATS*1 and *PTPN*14 genes, provides clear evidence of the involvement of this signaling cascade in BCC, adding BCC to the broad group of human cancers with a deregulated Hippo–YAP axis. In addition, three candidate genes, *MYCN*, *PPP*6*C,* and *STK*19, known to be associated with melanoma development, might be considered as new BCC driver genes. Of note, emerging reports are showing that somatic, non–coding mutations within promoter regions of *TERT* and *DPH*3*-OXNAD*1 genes are common in all types of skin cancer, including BCC. All these evidences demonstrate a complex genetic network of cancer-related genes and different pathways contributing to BCC carcinogenesis, supporting a heterogeneous genetic origin. Understanding the molecular genetics of BCC tumorigenesis might have a major impact on the development of novel target therapies to be used as single agent or in combination, allowing us to enhance treatment efficacy and overcome tumor resistance.

BCC tumors display great variability in morphology, aggressiveness, and response to treatment, and there is an ongoing debate about whether the clinico-pathological heterogeneity of BCC is related to distinct molecular subtypes. The integration of molecular genetic findings with the clinico-pathological features of the tumors might lead to a better BCC classification that definitely will change diagnostic and treatment algorithms.

## Figures and Tables

**Table 1:** Frequency of mutations and loss of heterozygosity (LOH) in cancer-related genes across published studies in basal cell carcinoma (BCC).

Gene	Nr. of Samples Analyzed	Mutations (%)	LOH (%)	References
*PTCH1*	37	32.4	24.3	[12]
	55	-	66.7	[51]
	26 ^a^	11.8	38.2	[15]
	24	54.2	-	[11]
	15	40.0	53.3	[18]
	31	54.8	43.5	[52]
	42	66.7	52.6	[19] *
	14 ^b^	64.3	92.8	[49]
	12 ^b^	8.3	40.0	[50]
	12	75.0	41.7	[48]
	293 ^c^	73.0	55.0	[22] *
*TP53*	14	50.0	na	[57]
	18 ^d^	61.1	5.5	[51]
	27	56.0	na	[58]
	20	35.0	na	[59]
	24	45.8	na	[11]
	15	33.0	-	[18]
	50 ^e^	66.0	na	[60]
	98 ^f^	37.7	na	[60]
	42	40.5	7.9	[48]
	30	20.0	na	[61]
	12	66.7	na	[19]
	293	61.0	17.0	[22] *
*SMO*	47	6.38	na	[14]
	42	9.5	na	[48]
	293	20.0	na	[22] *
*SUFU*	42	2.4	na	[48]
	293	8.0	5.0	[22] *
*NOTCH1*	12	50.0	na	[19] *
	293	29.0	2.7	[22] *
*NOTCH2*	12	66.7	na	[19] *
	293	26.0	na	[22] *
*LATS1*	293	16.0	4.0	[22] *
*LATS1*	293	12.0	5.0	[22] *
*PPP6C*	293	15.0	46.0	[22] *
*STK19*	293	10.0	na	[22] *
*MYCN*	293	30.0	na	[22] *
*ARID1A*	293	26.0	3.0	[22] *
*PTPN14*	293	22.0	5.0	[22] *
*CASP8*	293	11.0	3.0	[22] *
*CSMD1*	12	91.7	na	[19] *
*DPP10*	12	75.0	na	[19] *
*CSMD2*	12	66.7	na	[19] *
*CSMD3*	12	58.3	na	[19] *
*PREX2*	12	58.3	na	[19] *
*DCC*	12	50.0	na	[19] *
*GRIN2A*	12	50.0	na	[19] *
*TERT-promoter*	32	56.2	na	[23]
	42 ^g^	73.8	na	[24]
	196 ^h^	38.8	na	[25]
	137	65.0	na	[26]
*DPH3/OXNAD1 promoter*	137	41.6	na	[26]

^a^ 26 sporadic basal cell carinomas (BCCs) and 8 BCCs from patients with Gorlin Syndrome; ^b^ 6 sporadic BCCs and 6 BCCs from patients with with Gorlin Syndrome; ^c^ 263 sporadic BCCs and 30 BCCs from patients with Gorlin Syndrome; ^d^ subset of BCCs from sun-exposed areas; ^e^ aggressive BCCs; ^f^ non-aggressive BCCs; ^g^ 23 sporadic BCCs and 19 BCCs from patients with Gorlin Syndrome; ^h^ 94 from non-X irradiated patients and 102 from X-irradiated patients; * only genes with a mutation frequency higher than 10% have been reported. LOH, copy loss of heterozygosity; na, not applicable.
